# Impact of vegetable crop agriculture on anopheline agressivity and malaria transmission in urban and less urbanized settings of the South region of Cameroon

**DOI:** 10.1186/s13071-015-0906-2

**Published:** 2015-05-28

**Authors:** Patrick Ntonga Akono, Jean Arthur Mbida Mbida, Calvin Tonga, Philippe Belong, Odette Etoile Ngo Hondt, Gaëlle Tamdem Magne, Marie Florence Peka, Leopold Gustave Lehman

**Affiliations:** Laboratory of Animal Biology, Department of Animal Biology, Faculty of Science, University of Douala, P.O. Box 2701, Douala, Cameroon; Higher Teacher training college, University of Yaoundé I, P.O. Box 812, Yaoundé, Cameroon; Laboratory of Zoology, Department of Biology and Animal Physiology, Faculty of Science, University of Yaoundé I, P.O. Box 812, Yaoundé, Cameroon

**Keywords:** Vegetable crop agriculture, Hydro-agricultural lands, Anopheles, Malaria, Less urbanized setting, Urban setting, Cameroon

## Abstract

**Background:**

The use of inland valley swamps for vegetable crop agriculture contributes to food security in urban and less urbanized settings in Africa. The impact of this agriculture on aggressive mosquitoes’ diversity and malaria transmission in central Africa is poorly documented. This study is aimed at assessing the impact of vegetable crop agriculture on these entomological parameters in urban and less urbanized settings of the forest area, south of Cameroon.

**Methods:**

The human bait technique was used for the capture of aggressive mosquitoes from January to December 2012. For three consecutive days each month, captures were performed on volunteers in hydro-agricultural and river bank sites of Akonolinga and Yaoundé. Physico-chemical characteristics of mosquito breeding sites were recorded. Molecular alongside morpho-taxonomic techniques were used for the identification of mosquito species; ELISA test was used to reveal *Plasmodium falciparum* infected mosquitoes through the detection of CSP. Mosquito diversity, aggressivity and malaria transmission in sites and settings were determined and compared.

**Results:**

Biting rates were higher in hydro-agricultural sites of less urbanized and urban settings (31.8 b/p/n and 28.6 b/p/n respectively) than in river banks sites (6.83 b/p/n and 3.64 b/p/n respectively; *p* < 0.0001). Physico-chemical parameters of breeding sites were not fundamentally different. Five anopheline species were identified; *An. gambiae*, *An. funestus* s.s., *An. moucheti* s.s., *An. hancocki* and *An. nili* s.s. In hydro-agricultural sites 2 species were captured in the urban setting versus 4 in the less urbanized setting, meanwhile in river bank sites, 3 species were captured in the urban setting versus 4 species in the less urbanized setting. *An. nili* s.s. was found in river banks only. *An. hancocki* was not found to insure *Plasmodium falciparum* Welch transmission. EIR in hydro-agricultural sites varied from 1.86 ib/p/n (urban area) to 2.13 ib/p/n (less urbanized area) with higher rates in April/May and August. Overall, EIR was higher in less urbanized areas (*p* < 0.0001) but the difference was nullified with the practice of vegetable crop agriculture (*p* = 0.2).

**Conclusion:**

These results highlight the need for specific preventive measures that take into account the ecological peculiarities related to vegetable crop agriculture on hydro-agricultural lands, in order to protect inhabitants from malaria.

## Background

Within the past 20 years, demographic growth has been one of the most striking phenomena in tropical countries, with subsequent rural exodus [1]. From less than 500,000 inhabitants each in 1970, the populations of Douala and Yaoundé, the two main cities in Cameroon are nowadays higher than 2,500,000 inhabitants [2]. Population growth in urban settings has resulted in increased demand for food supply. A solution to the problem has been the development of inland valley swamps into hydro-agricultural lands. In Cameroon, inland valley swamps in urban settings are state properties. For some years, local authorities have liberalized the use of these bottomlands. Thus the development of inland valley swamps in Douala and Yaoundé into hydro-agricultural lands leading to an increase in crop production. This is also the case in less urbanized settings such as Foumbot, Obala and Akonolinga, which supply highly urbanized cities in vegetables and food crops. Pea nuts, maize, tomatoes, pineapple and vegetables are some of the products of hydro-agricultural lands of the southern part of Cameroon; they are highly appreciated for their nutritional value. Though hydro-agricultural lands contribute to food security by increasing crop production, they may have a deep influence on malaria transmission, due to the creation of breeding sites for mosquitoes [3].

In many urban settings of East and West Africa, these hydro-agricultural lands serve mostly for the cultivation of rice. The impact of rice paddies on mosquito fauna has been assessed in many towns, their influence on malaria transmission being variable [4–8]. On the reverse, in most Central African urban or less urbanized settings as is the case in Cameroon, hydro-agricultural lands are mostly occupied by vegetable crops. Depending on the level of urbanization, the development of hydro-agricultural lands may cause important ecological changes with possible effects on malaria vectors and malaria transmission dynamics. However, data on the impact of the development of inland valley swamps into hydro-agricultural lands on mosquito fauna and malaria transmission are scarce in the literature. In Yaoundé and Akonolinga, South of Cameroon, vegetable crop farms occupy 40 and 130 ha respectively; to date, no entomological data is available for these areas.

This study reports on the development of inland valley swamps into hydro-agricultural lands for vegetable crops agriculture and its impact on mosquito fauna and malaria transmission in Akonolinga and Yaoundé, equatorial areas of degraded forest South of Cameroon, with different levels of urbanization. This will help for the design of better vector control strategies.

## Methods

### Description of the study area

**Fig. 1:**
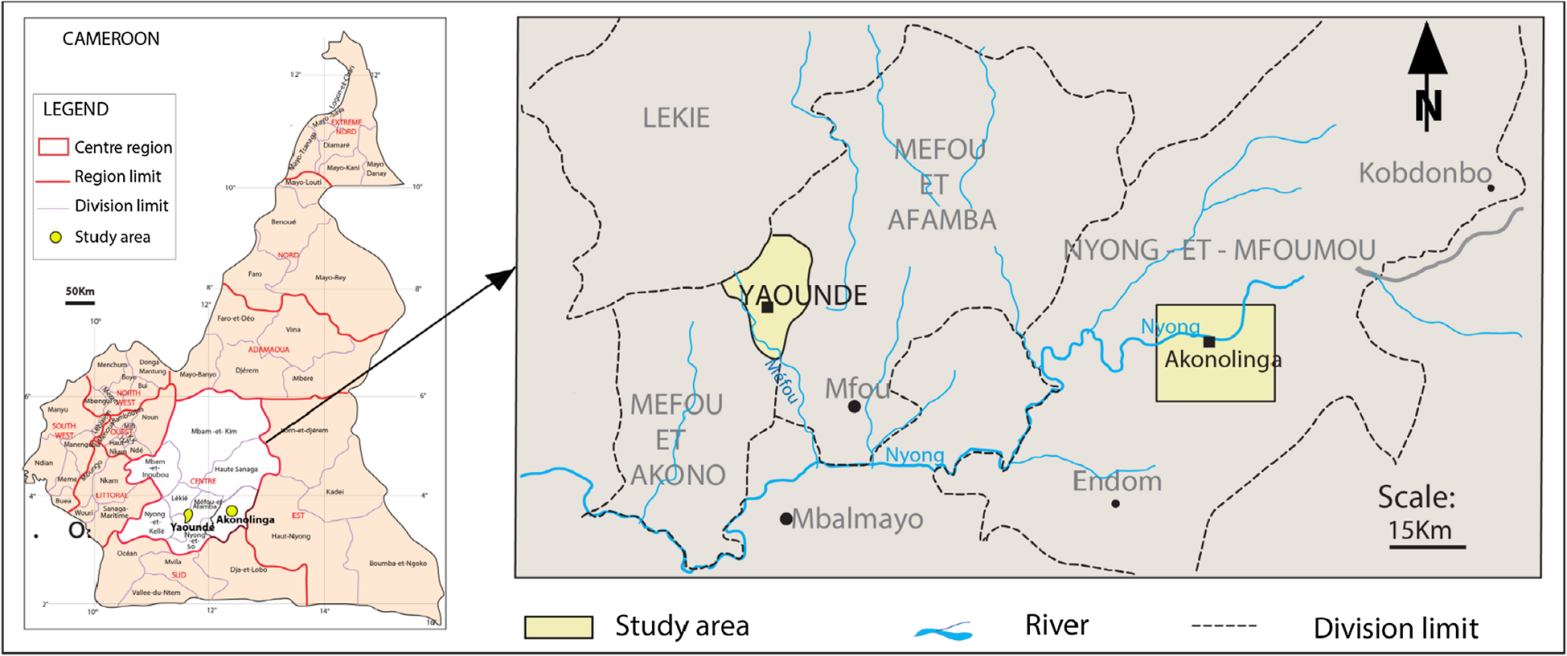
Map of the study area

The study was conducted in Akonolinga (3°46’N, 12°15’E) and Yaoundé (3° 51’N, 11°30’E), in the forest area South of Cameroon. The climate is equatorial (Guinean type) with two rainy seasons (September to November and March to June) alternating with two dry seasons (December to February and July to August) [9]. Rainfalls are abundant and the mean annual temperature was 25. 7 °C in Akonolinga and 26.06 °C in Yaoundé. Yaoundé is a city of 2.5 million inhabitants with highly degraded vegetation as a consequence of urbanization. Akonolinga is a less urbanized setting located a hundred kilometers East of Yaoundé and less populated (74,000 inhabitants) [2]. The hydrographic network is dense and consists in many streams and swampy inlands. The river Mefou runs through the southern part of Yaoundé meanwhile the river Nyong runs through Akonolinga, East to West (Fig. 1). For more than two decades, swampy inland valleys have gradually been transformed into hydro-agricultural lands in order to meet food needs of the growing populations. These lands generally receive one crop cycle in the year, from November to March, the period during which they receive appropriate irrigation.

In each of the towns, entomological surveys were carried out in two different ecological sites: a vegetable crop cultivation site and a river bank site. Nkolbisson in Yaoundé and Nlong-assi in Akonolinga are crossed by river Mefou and river Nyong respectively meanwhile Ngousso in Yaoundé and Loum in Akonolinga are characterized by large hydro-agricultural surfaces. The distance between the 2 sites is 6 km in Akonolinga and 7 km in Yaoundé.

### Study design and ethical consideration

This longitudinal study was carried out for 12 months, from January to December 2012 with the permission of Senior Divisional Officers of the areas and the consent of owners of the houses in which the catches were performed.

### Mosquito sampling

Sampling was done for three consecutive days each month, consisting in the capture of female mosquitoes by landing catch on human volunteers. Prior to this activity, the volunteers were immunized against yellow fever; they also received antimalarial chemoprophylaxis. Catches were performed in six different residences situated nearby main breading sites on river banks or hydro-agricultural lands. In each of the residences, 2 teams of 3 capturers were working in turn, in two shifts; one from 6 p.m. to midnight and the other from midnight to 6 a.m. Overall, 648 volunteers participated in each site throughout the 36 nights during which captures were performed. Mosquitoes were morphologically identified to species following the taxonomic keys [10–12]. Specimen were then stored in silicagel containing Eppendorf tubes at −20 °C, for subsequent laboratory analysis.

### Laboratory analysis

Infection rate was determined through the detection of the circumsporozoite protein (CSP). This protein found on the surface of the sporozoite stage of *Plasmodium* was searched for, in the head and thorax of female *Anopheles* mosquitoes through the ELISA technique [13, 14]. Species and molecular forms of *An. gambiae* complex as well as species of *An. funestus, An. moucheti* and *An. nili* groups were identified by the PCR-based method [15–18].

### Physicochemical analysis of water from larval habitats

A landing net was use for the capture of aquatic macro-invertebrates. Orion 5-star portable Multiparameter (Thermo-Scientific) was used to determine the physico-chemical parameters of breeding sites such as pH, la conductivity (μS/cm), salinity, TDS (mg/L), turbidity (NTU) and dissolved oxygen (%). Concentrations of sulfates, nitrates and phosphates were measured with a spectrophotometer HCHDR 2000 while Flame Atomic Absorption Spectrometry (FAAS) was used for determining calcium and magnesium concentrations. A thermometer was used to measure water temperature.

### Data analysis

Biting rate was expressed as the average number of bites received per person per night of collection. Sporozoitic index was expressed as the proportion of mosquitoes found to contain circumsporozoite antigens by ELISA. Entomological Inoculation Rate (EIR) was expressed as the number of infecting bites per person per night or year (ib/p/n or ib/p/y) and calculated as the product of the sporozoitic index and the biting rate. Statistical analyses were performed with the SPSS (Version 19.0 for Windows, SPSS Inc., Chicago, IL, U.S.A.). The Student *t*-test was used to compare Entomological Inoculation Rates (EIR) and mean biting rates in the study sites. Spearman test was used to assess the correlation between rainfall and mosquito biting rate as well as the correlation between rainfall and EIR. The level of significance was set at α = 0.05.

## Results

### Biting rate and effect of rainfall

#### Hydro-agricultural sites

A total of 18,530 female mosquitoes were captured in the Ngousso neighborhood, Yaoundé. The mean biting rate recorded was 28.6 b/p/n (10,439 b/p/y). Anopheles mosquitoes contributed for 97.2 % of the total aggressivity. *An. gambiae* (the only species of the Gambiae complex out of 1500 specimens successfully identified by PCR technique) was the most aggressive species (17.4 b/p/n or 6351 b/p/y) followed by *An. funestus* s.s. (the only species of the Funestus group out of 600 specimens successfully identified by PCR technique) (10.4 b/p/n or 3796 b/p/y) (Table 1).

**Table 1:** Mosquito abundance, biting rates, sporozoitic index and EIR in urban and less urbanized settings

	Mosquito species	Abundance^a^		Biting rates^d^ (b/p/n)		Sporozoitic index^e^ (%)		EIR^d^ (ib/p/n)	
		HA^b^ site	RB^c^ site	HA site	RB site	HA site	RB site	HA site	RB site
Urban settings (Yaoundé)	*An. gambiae*	11,268	1744	17.4	2.7	1.7	8.3	1.39	1.13
	*An. funestus* s.s.	6723	44	10.4	0.07	0.9	6.8	0.47	0.007
	*An. nili* s.s.	-	374	-	0.6	-	5	-	0.03
	Culicinae	539	181	0.8	0.27	-	-	-	-
	Overall	18,530	2343	28.6	3.64	1.4	6.7	1.86	1.167
Less urbanized settings (Akonolinga)	*An. gambiae*	9911	1573	15.3	2.4	1.8	7.05	1.32	0.9
	*An. funestus* s.s.	7185	746	11.1	1.2	1.5	8.2	0.8	0.5
	*An. moucheti* s.s.	128	1778	0.2	2.7	5.5	7.06	0.01	0.93
	*An. hancocki*	28	16	0.04	0.03	-	-	-	-
	Culicinae	3382	323	5.2	0.5	-	-	-	-
	Overall	20,634	4436	31.8	6.83	1.8	7.2	2.13	2.33

^a^Abundance: Within 648 man-night captures

^b^HA: Hydro-Agricultural

^c^RB: River Bank

^d^Biting rates and EIR are calculated over the year

^e^ELISA test was performed on part of the total number of Anopheles mosquitoes captured

In Loum (Akonolinga), a less urbanized setting, a total of 20,634 females were captured. Mean biting rate was 31.8 b/p/n or 11,607 b/p/y. Anopheles mosquitoes contributed for 83.6 % of the total aggressivity. *An. gambiae* (the only species of the Gambiae complex on the 1500 specimens successfully identified by PCR technique) (15.3 b/p/n or 5584.5 b/p/y) was the most aggressive species, followed by *An. funestus* s.s. (the only species of the Funestus group out of 700 specimens successfully identified by PCR technique) (11.1 b/p/n or 4051.5 b/p/y). *An. hancocki* was the less aggressive species (0.04 b/p/n or 14.6 b/p/y) (Table 1).

**Fig. 2:**
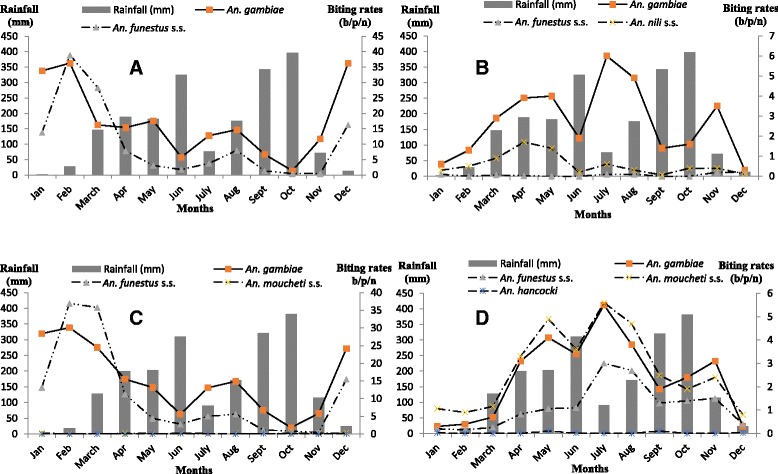
Variation of biting rates in relation with rainfall and sites characteristics (**a**: Hydro-agricultural site in urban setting; **b**: River bank site in urban setting; **c**: Hydro-agricultural site in less urbanized setting; **d**: River bank site in less urbanized setting)

A negative correlation was found between biting rates and rainfall (*r* = −0.76, *p* = 0.003 in Ngousso; *r* = −0.7, *p* = 0.01 in Loum). But for few exceptions, biting rates followed similar trends in the two neighborhoods, reaching their maximum during the January/February period (long dry season) and the July/August period (short dry season) (Fig. 2a and c).

#### River bank sites

Overall, 2343 female mosquitoes were captured in the Nkolbisson neighborhood (Table 1). The mean biting rate recorded was 3.64 bites/person/night (b/p/n) or 1328.6 bites/person/year (b/p/y). *An. gambiae* (the only species of the Gambiae complex out of 500 specimen successfully identified by the PCR technique) was the most aggressive (2.7 b/p/n or 985 b/p/y), followed by *An. nili* s.s. (the only species of the Nili group out of 300 specimen successfully identified by PCR technique) (0.6 b/p/n or 219 p/h/an b/p/y). The Culicinae were the less aggressive (0.27b/p/n or 98. 55 b/p/y) (Table 1).

In the Nlong-assi neighborhood, a less urbanized setting, a total of 4436 female mosquitoes were captured. The mean biting rate recorder was 6.83 b/p/n (2493 b/p/y). Anopheles mosquitoes contributed for 92.6 % of the total aggressivity. *An. moucheti* s.s. (the only species of the Moucheti group out of 1000 specimen successfully identified by PCR technique) appeared to be the most aggressive species (2.7 b/p/n or 985.5 b/p/y) followed by *An. gambiae* (the only species of the Gambiae complex out of the 700 specimens successfully identified by PCR technique) (2.4 b/p/n or 876 b/n/y). *An. hancocki* was the less aggressive species (0.03 b/p/n or 10.95 b/p/y) (Table 1).

But for few exceptions, seasonal variations followed similar trends in the two neighborhoods. Biting rates were higher during the short rainy season (March to June) and short dry season (July to August); they were lower during the long dry season (September to November) and the long rainy season (December to February) (Fig. 2b and d).

#### Comparison of biting rates in river bank and agricultural sites

In Yaoundé, urban setting, the mean daily biting rate recorded throughout the study period was significantly higher in the hydro-agricultural sites (28.6 b/p/n versus 3.64 b/p/n in the river bank site, *p* < 0.0001).

In Akonolinga, less urbanized setting, the means daily biting rate recorded throughout the study period was also significantly higher in the hydro-agricultural site (31.8 b/p/n versus 6.83 b/p/n in the river bank site, *p* < 0.0001).

As shown in Table 2, the global mean biting rate recorded in urban settings (16.12 b/p/n) and less urbanized settings (19.31 b/p/n) were not statistically different (*p* = 0.131), though higher in less urbanized settings.

**Table 2:** Comparison of biting rates and EIR in study sites

		Urbanization level		
		Less urbanized setting	Urban setting	*p*-value
Biting rate (b/p/n)	HA^a^ sites	31.8	28.6	0.48
	RB^b^ sites	6.83	3.64	<0.0001
	*p*-value	<0.0001	<0.0001	-
	Overall mean	19.315	16.12	0.131
EIR (ib/p/n)	HA sites	2.13	1.86	0.2
	RB sites	2.33	1.167	<0.0001
	*p*-value	0.02	0.008	-
	Overall mean	2.23	1.51	<0.0001

^a^HA: Hydro-Agricultural

^b^RB: River Bank

### Infectivity of vectors

#### Hydro-agricultural sites

**Fig. 3:**
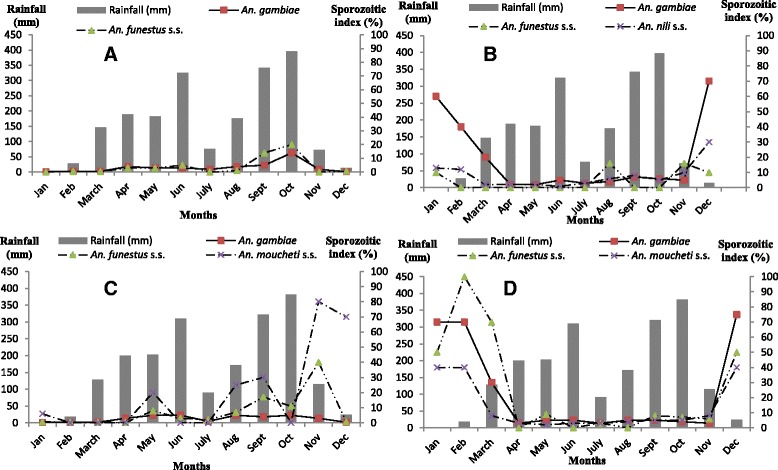
Variation of sporozoitic index in relation with rainfall and sites characteristics (**a**: Hydro-agricultural site in urban setting; **b**: River bank site in urban setting; **c**: Hydro-agricultural site in less urbanized setting; **d**: River bank site in less urbanized setting)

Overall 3589 female anopheles mosquitoes captured in Ngousso (Yaounde, urban setting) were submitted to the ELISA test for CSP. Mean sporozoitic index recorded in this site were 1.7 % and 0.9 % for *An. gambiae* and *An. funestus* s.s. respectively (Table 2). Infected *Anopheles gambiae* females were found throughout the year. *An. funestus* s.s. was absent in January, July and November (Fig. 3a).

A total of 3539 female anopheles mosquitoes captured in Loum (Akonolinga, less urbanized setting) were submitted to the ELISA test for CSP. Mean sporozoitic index recorded in this site were 1.8 %, 1.5 % and 5.5 % for *An. gambiae*, *An. funestus* s.s. and *An. moucheti* s.s. respectively (Table 1). Infected females were found throughout the year but for *An. moucheti* s.s. that was found in January, May, August, September, November and December (Fig. 3c).

#### River bank sites

From Nkolbisson (Yaounde, urban setting), 765 female anopheles mosquitoes were submitted to the ELISA test for CSP. Mean annual sporozoitic index recorded in this site were 8.3 %, 5 % and 6.8 % for *An. gambiae*, *An. nili* s.s. and *An. funestus* s.s. respectively (Table 1). Infected females were found throughout the year but for *An. funestus* s.s. that was found only in January, August, November and December only (Fig. 3b).

A total of 813 female anopheles mosquitoes captured in Nlong-assi (Akonolinga, less urbanized setting), were analysed for CSP through the ELISA test. Mean annual sporozoitic index were 7.05 %, 8.2 % and 7.06 % for *An. gambiae*, *An. funestus* s.s. and *An. moucheti* s.s. respectively (Table 1). Infected female anopheles mosquitoes were captured throughout the year but for *An. funestus* s.s. that was absent in April, June and August (Fig. 3d).

### Entomological inoculation rate (EIR) and malaria transmission

#### Hydro-agricultural site

**Fig. 4:**
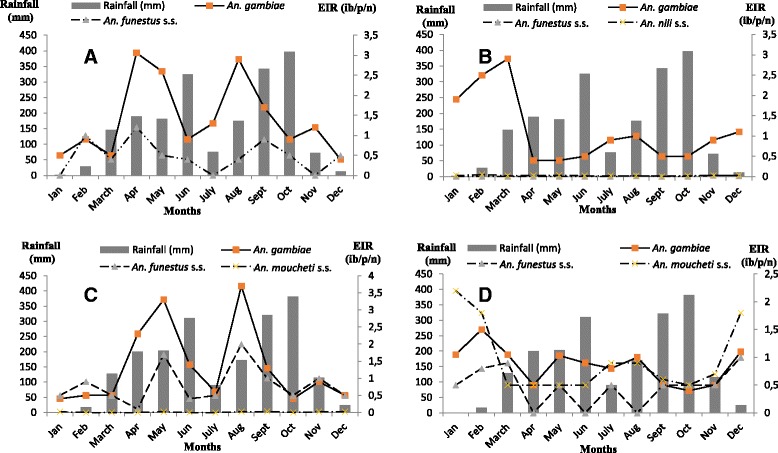
Variation of EIR in relation with rainfall and sites characteristics (**d**: Hydro-agricultural site in urban setting; **b**: River bank site in urban setting; **c**: Hydro-agricultural site in less urbanized setting; **a**: River bank site in less urbanized setting)

Malaria transmission is perennial in Loum, insured by three out of the four anopheline species identified in this site, *An. gambiae*, *An. funestus* s.s., and *An. moucheti* s.s.. *An. gambiae* is the main malaria vector in this site (1.32 ib/p/n or 481.8 ib/p/y), being responsible for 61.9 % of the transmission (Table 1). Transmission due to these species is high in May and August. The number of infective mosquito bites received per annum by an inhabitant of this area is at 777 (Fig. 4c).

Malaria transmission is also perennial in Ngousso, insured by the two anopheline species identified in this site, *An. gambiae*, and *An. funestus* s.s.. *An. gambiae* is the main malaria vector in this site (1.39 ib/p/n or 507.4 ib/p/y), being responsible for 75.07 % of malaria transmission (Table 1). Transmission due to this species is high in April, May and August (Fig. 4d). The number of infective mosquito bites received per annum by an inhabitant of this area is estimated at 679.

#### River bank sites

In Nlong-assi, malaria transmission is perennial, insured by *An. gambiae*, *An. funestus* s.s., and *An. moucheti* s.s. *An. moucheti* s.s. (0.93 ib/p/n or 339.4 ib/p/y) and *An. gambiae* (0.9 ib/p/n or 328 ib/p/y) are the major malaria vectors in this site, being responsible for 78.5 % (Table 1). Transmission is higher in the months of December and March (long dry season) (Fig. 4a). The number of infective mosquito bites received per annum by an inhabitant of this area is estimated at 851.

In Nkolbisson, malaria transmission is perennial, insured by three anopheline species: *An. gambiae*, *An. nili* s.s. and *An. funestus* s.s. *An. gambiae* (1.13 ib/p/n or 412.4 ib/p/y) is the main malaria vector in this site, being responsible for 97.1 % of malaria transmission (Table 1). Malaria transmission is high in December and March (long dry season) (Fig. 4b). The number of infective mosquito bites received per annum by an inhabitant of this area is estimated at 426.

#### Comparison of EIR

In the urban setting, the overall mean daily EIR recorded in the river bank site (Nkolbisson) was 1.167 ib/p/n versus 1.86 ib/p/n in the hydro-agricultural site (Ngousso). Overall mean daily EIR was significantly lower in the river bank site than in the hydro-agricultural site (*p* = 0.008).

In the less urbanized setting, the overall mean daily EIR recorded in the river bank site (Nlong-assi) was 2.33 ib/p/n versus 2.13 ib/p/n in the hydro-agricultural site (Loum). In this less urbanized setting, the overall mean daily EIR was significantly higher in the river bank site than in the hydro-agricultural site (*p* = 0.02).

As shown in Table 2, the overall mean daily EIR recorded in Yaoundé, urban area was 1.51 ib/p/n, significantly lower than in Akonolinga, less urbanized area where it was 2.23 ib/p/n (*p* < 0.0001).

#### Physico-chemical parameters

Physico-chemical parameters of prospected breeding sites are reported in Table 3. Breeding sites in hydro-agricultural and river bank sites of urbanized and less urbanized settings were acidic (pH between 5.01 and 6.9) and well oxygenated (dissolved oxygen between 8.5 and 9.7 mg/L). They contained few dissolved organic matter (TDS between 50.6 and 110.3 mg/L). Concentrations of nitrates, sulfates and phosphates were low. These parameters are adequate for the development of anopheles larvae. However, river bank breeding sites contained more aquatic plants, various species of fishes and aquatic macro-invertebrates; they also had lower temperatures than hydro-agricultural breeding sites.

**Table 3:** Physicochemical parameters of mosquito breeding sites

Parameters	Urban setting (Yaoundé)				Less urbanized setting (Akonolinga)			
	Hydro-agricultural site		River site		Hydro-agricultural site		River site	
	Mean ± SE	Range	Mean ± SE	Range	Mean ± SE	Range	Mean ± SE	Range
Dissolved oxygen	7.8 ± 0.7	7.3–8.3	9.7 ± 1.1	8.9–11.2	9.2 ± 0.5	6.1–7.8	8.5 ± 0.7	6.3–9.2
pH	5.01 ± 0.5	5.7–6.4	6.3 ± 0.7	4.9–6.3	6.7 ± 1.1	5.2–7.3	6.9 ± 0.4	6.2–8.1
Salinity (%)	0.09 ± 0.01	0.08–0.1	0.07 ± 0.01	0.05–0.1	0.07 ± 0.01	0.06–0.09	0.1 ± 0.01	0.08–0.2
Conductivity (μS/cm)	294.7 ± 8.9	275.5–304.5	305.4 ± 5.7	277.3–325.1	247.3 ± 7.2	228.4–269.5	294.2 ± 5.4	250.3–335.5
Water temperature (°C)	27.5 ± 2.3	22.2–30.4	22.2 ± 3.1	21.3–28.2	28.4 ± 2.9	24.6–32.1	22.7 ± 1.8	24.3–29.5
Total dissolved solids (mg/L)	50.6 ± 2.6	60.4–70.2	110.3 ± 4.2	97.3–124.2	71.4 ± 1.2	64.5–78.3	97.9 ± 2.4	86.8–107.4
Turbidity (NTU)	31.2 ± 2.7	27–35	27 ± 1.7	24–31	35 ± 2.3	30–40	28 ± 2.1	22–34
Sulfate (mg/L)	3.1 ± 0.3	2.7–3.5	0 ± 0	0–0	2 ± 0.1	1.7–2.3	0.2 ± 0.01	0.008–0.4
Phosphate (mg/L)	0.12 ± 0.03	0.08–0.16	0.17 ± 0.02	0.09–0.16	0.2 ± 0.01	0.08–0.7	0.05 ± 0.001	0.02–0.08
Nitrate (mg/L)	2.3 ± 0.2	2.0–2.6	2.4 ± 0.1	1.8–2.8	1.9 ± 0.1	1.5–2.3	2.1 ± 0.3	1.9–2.3
Magnesium (mg/L)	30 ± 2.8	25–33	27 ± 1.8	23–31	33 ± 1.4	27–36	21 ± 0.9	18–25
Calcium (mg/L)	94 ± 8.9	90–98	115 ± 7.9	95–130	87 ± 1.3	79–98	112 ± 2.6	97–119
Aquatic plants	(+)	-	(+++)	-	(+)	-	(+++)	-
Fish	(−)	-	(+++)	-	(−)	-	(+++)	-
Aquatic invertebrates	(−)	-	(+++)	-	(−)	-	(+++)	-

(+++) = very abundant; (+) = scarce; (−) = absent - = No data available

## Discussion

This study compares the anopheline diversity, and malaria transmission in river bank and hydro-agricultural sites in two settings of South Cameroon, Yaoundé and Akonolinga. Yaoundé is highly urbanized while Akonolinga is a less urbanized setting. Molecular techniques coupled with classical morpho-taxonomic methods showed that aggressive anopheline fauna in both settings is rich and varied. It consists of five Anopheles species of which 4 are known to be major malaria vectors in tropical Africa: *An. gambiae*, *An. funestus* s.s., *An. moucheti* s.s., and *An. nili* s.s. [19–22]. Anopheline diversity could be related with the presence of various types of breeding sites in the survey area. These breeding sites are different by the ecological and physico-chemical nature of the water they contain. Less urbanized settings recorded higher anopheles biting rates than urban settings. Being more natural, less urbanized settings may be more favorable for the proliferation of mosquitoes of the genus *Anopheles.* On the other hand, hydro-agricultural sites recorded higher biting rates. Similar results were obtained in several cities of Africa, highlighting the prolificacy of mosquito breeding sites in hydro-agricultural areas of urban settings [23–25]. Though physico-chemical characteristics (dissolved oxygen, TDS, pH, concentrations of nitrates, sulfates, chlorides, potassiums and phosphates) are similar in hydro-agricultural and river bank sites, there is a difference in mosquito abundance that might be related to the temperature of the water found in breeding sites [26], and the presence or not of aquatic plants as well as aquatic macro-invertebrates and vertebrates; these animals may behave as predators for mosquito larvae. Hydro-agricultural sites offer highly diversified ecosystems with multiple permanent breeding sites resulting from ridge furrows made during the planting season. The permanence of these breeding sites owes to the irrigation system set by farmers. Mosquito abundance recorded here may be due to the absence of shading plants resulting in highly sunlit breeding sites, favorable for the development of *An. gambiae* larvae, known to be a heliophilic species [19, 27]. Temperature between 28 and 30 °C may be favorable for rapid development of *An. gambiae* larvae [28]. The proliferation of anopheles mosquitoes in hydro-agricultural sites may also be caused by the use of fertilizers and chemicals by farmers. These chemicals may eliminate potential predators, therefore favoring the proliferation of *An. gambiae* larvae that are resistant to many of these chemicals [29]. On the reverse, river bank sites are rich with aquatic plants, mostly *Pistia sp.* These plants retain sunlight, resulting in lower temperature of breeding sites. Low temperatures are known to slow down the growth of the micro-organisms *An. gambiae* larvae feed on in their natural environment [30]. The low abundance of anopheles mosquitoes may also be due to the presence of larvivorous fish species such as *Gambusia affinis* (Guppy) found in river bank sites of less urbanized settings. A study carried out in fish paddies in India have shown that *Gambusia affinis* (Guppy) can cause up to 88 % reduction of the population of anopheles larvae. [31]. Moreover, presence of tadpoles, aquatic insects of the order Coleoptera and Hemiptera could also contribute to the reduction of anopheles larvae in river banks breeding sites. These animals exert predation on anopheles larvae or compete for food [32].

Fluctuations of aggressive anopheline mosquito density appear to depend on the regularity of crop irrigation, sunshine and rainfall. Anopheline densities are highest from December to March (long dry season); this may be due to the fact that during the dry season, most of the breeding sites dry up, forcing adult female Anopheles mosquitoes to lay their eggs in hydro-agricultural sites that are irrigated. During this period, a fluctuation in aggressive *Anopheles gambiae* and *Anopheles funestus* s.s. densities was observed over time. From December to January the density of aggressive *Anopheles gambiae* was higher than that of *An. funestus* s.s. A reverse tendency was observed from February to March. Similar trends were observed in rice paddies in Bouaké, Ivory Coast [6]. This could be explained by the fact that during the planting season (November-December), mosquito breeding sites are very sunlit, which promotes the proliferation of *An. gambiae* [19, 27]. In the months of February and March, the plants have grown considerably and have shaded the breeding sites rendering them more suitable for *An. funestus* s.s. larvae known to be heliophobic [27]. The months of September and October are characterized by heavy rains in the study area (great rainy season). Frequent rainfall results in the leaching of most breeding sites; hence the low density of aggressive Anopheles mosquitoes observed during this period in the hydro-agricultural sites.

Five anophelines species were captured in river bank sites, despite their low densities. These are: *An gambiae*, *An funestus* s.s., *An moucheti* s.s., *An nili* s.s. and *An hancocki. An moucheti* s.s. larvae prefer permanent slow moving water bodies, with plants such as *Pistia* sp*.* [33]. The capture of this species in Nlong-assi (Akonolinga) can be explained by the fact that river Nyong is a slow running river, overgrown in places by plants, including *Pistia* sp. *An. hancocki* had long been reported in Ayos, a town not far from our study site [34]. The presence of this anophelines species in Akonolinga may result from their survival to the deep ecological upheavals that have been occurring in this area since 1947. On the reverse, river Mefou in Yaoundé is a rapid river. Capturing *An. nili* s.s. in the houses near the river may be due to the fact that larvae of this species develop in the vicinity of fast flowing rivers [35]. The capture of adult female *An. gambiae* s.s. and *An. funestus* s.s. in river bank sites can be explained by the presence of multiple small water collections overgrown or not with plants, more or less sunlit, in the vicinity of the river. These small water bodies are generally formed in cattle footprints resulting from their occasional grazing at the river bank.

Fluctuations in aggressive anopheline mosquito density in river banks sites could depend on rainfall and the flow of the river. Variations in aggressive anopheline mosquito density in these sites follow similar trend. They are low during the long dry season and the long rainy seasons, but high in the short dry and the short rainy seasons. The low mosquito density recorded during the long dry season could be consecutive to the drying up of temporary water collections in the vicinity of the river. Moreover, lower densities of aggressive *An. moucheti* s.s. observed in these periods in the river bank site in Akonolinga may be due to weeding activities performed on the river every year in these periods, in order to combat invasive weeds such as *Eichhornia crassipes* and *Pistia* sp. This certainly disrupts the habitat of *An. moucheti* s.s larvae. During the long rainy season, the decrease in anopheline density may result from the aggressive leaching of breading sites by floods, as the level of rivers increase gradually with rainfall and drain the sites. High anopheline densities observed from March to August could be the result of scarce and light rainfall that favor the creation of water ponds, suitable for the development of the pre-imaginal stages of anopheles mosquitoes.

Circumsporozoite ELISA test performed on adult female anopheles mosquitoes showed that 4 out of the 5 species of anopheles captured ensure the transmission of malaria in study sites; these are *An. gambiae*, *An. funestus* s.s., *An. moucheti* s.s. and *An. nili* s.s. Overall, *An. gambiae* appears to be the major malaria vector in hydro-agricultural sites and the river Mefou bank site. This observation is consistent with those in most equatorial areas of the South of Cameroon where *An. gambiae* is known to be the major malaria vector [36]. *An. gambiae* is preceded in this role by *An. moucheti* s.s. in the banks of the river Nyong. This species has been reported by many authors as the major malaria vector in different ecological settings crossed by major rivers, in the southern area of Cameroon [33]. This result is however different from that recorded in the town of Ayos which is also crossed by river Nyong. In this locality, *An. gambiae* was identified as the major malaria vector [37]. The uprise of this species as the major malaria vector in Ayos might have been favored by the creation of breeding sites suitable for the development of *An. gambiae* during the construction of the Yaounde-Bonis road [37].

Malaria transmission follows similar trends in river bank sites and varies with seasons. Transmission starts from the beginning of the dry season in late November and remains very high during the second half of this season, with high infectivity of anopheles mosquitoes and lower biting rates. It decreases considerably during the short rainy season with an increase in the density of aggressive anopheles mosquitoes. This fluctuation may be explained by the fact that during the rainy season, the population turnover is higher, reducing the rate of infected anopheles in the general mosquito population. On the reverse, the renewal of the mosquito population is lower during the dry season, with subsequent increase in the rate of infected anopheles mosquitoes. However, in hydro-agricultural sites, the transmission is rather low from December to March (long dry season) and becomes relatively important in the months of April-May and August-September. The low transmission results from the renewal of the anopheles community observed during the long dry season with the irrigation of plants. This renewal is low from April to May and from August to September, increasing the rate of infected anopheles mosquitoes. Overall EIR was significantly higher in hydro-agricultural sites than in river bank sites and this was also observed in Yaoundé. Similar results were recorded by many researchers [23]. They found in Kumasi, Ghana, that EIR were higher in agricultural areas than non-agricultural areas, no matter the season. On the reverse, in the less urbanized setting of Akonolinga, overall EIR appeared to be lower in the hydro-agricultural site than in the river bank site. Thus, the development of inland valley swamps into hydro-agricultural lands has resulted in an increase in the anopheline biting rate and EIR in the urban area; in less urbanized areas, this resulted only in an increase of anopheline biting rate.

## Conclusions

These results call for African leaders to define new orientations aimed at reducing the creation of mosquito breeding sites in the course of the development of inland valley swamps into hydro-agricultural lands both in urban and less urbanized settings. Similar investigations should be conducted in many other African cities where vegetable crops are part of the urban landscape, in order to collect entomological baseline data needed for the design of effective control strategies, adapted to eco-climatic characteristics of each urban site.

## Abbreviations

b/p/n: Bites/person/night
CSP: Circumsporozoïtic protein
EIR: Entomological inoculation rate
ELISA: Enzyme linked immunobsorbent assay
ib/p/n: Infective bites/person/night
ib/p/y: Infective bites/person/year
PCR: Polymerase Chain Reaction
